# Supplementary material for: Identification and functional analysis of non-coding regulatory small RNA FenSr3 in Bacillus amyloliquefaciens LPB-18
Source: PeerJ. 2023 May 15;11:e15236. doi: 10.7717/peerj.15236 (PMC10194069; doi:10.7717/peerj.15236)
Supplement: Supplemental Information 4 [file peerj-11-15236-s004.zip › KO/CK-vs-T1_map/map03320.html]

KEGG PATHWAY: PPAR signaling pathway - Reference pathway


|  |  |
| --- | --- |
| **PPAR signaling pathway - Reference pathway** |  |

[
Pathway menu
| Organism menu
| Pathway entry
| Show description
| User data mapping
]

Peroxisome proliferator-activated receptors (PPARs) are nuclear hormone receptors that are activated by fatty acids and their derivatives. PPAR has three subtypes (PPARalpha, beta/delta, and gamma) showing different expression patterns in vertebrates. Each of them is encoded in a separate gene and binds fatty acids and eicosanoids. PPARalpha plays a role in the clearance of circulating or cellular lipids via the regulation of gene expression involved in lipid metabolism in liver and skeletal muscle. PPARbeta/delta is involved in lipid oxidation and cell proliferation. PPARgamma promotes adipocyte differentiation to enhance blood glucose uptake.

|  |  |  |
| --- | --- | --- |
| Reference pathway | 184% 150% 122% 100% 82% 67% 55% | 图片下载 |
